# Ozone-based interventions to mitigate *Pseudomonas paracarnis*-induced blue discoloration in fresh cheese

**DOI:** 10.1007/s10068-026-02237-2

**Published:** 2026-07-21

**Authors:** Juniel Marques de Oliveira, Laysha Neto de Paula Campos, Emanoel Henrique Fialho Ferreira, Letícia Elisa Rossi, Rafaela da Silva Rodrigues, Marcus Vinicius de Assis Silva, Ernandes Rodrigues de Alencar, Marcio Arêdes Martins, Mauricio de Oliveira Leite, Antônio Fernandes de Carvalho, Solimar Gonçalves Machado

**Affiliations:** 1 Department of Microbiology, Universidade Federal de Viçosa, Viçosa, MG 36570-900 Brazil; 2 Department of Food Technology, Av. Peter Henry Rolfs, S/N - Campus Universitário, Viçosa, MG 36570-900 Brazil; 3 Department of Agricultural Engineering, Universidade Federal de Viçosa, Viçosa, MG 36570-900 Brazil; 4 Department of Veterinary Medicine, Universidade Federal de Viçosa, Viçosa, MG 36570-900 Brazil

**Keywords:** Spoilage, Blue discoloration, Ozonated mist, Gaseous ozone, Non-thermal decontamination

## Abstract

Blue discoloration caused by *Pseudomonas* spp. remains a recurring issue in fresh cheeses. This study evaluated gaseous ozone and ozonated mist as two distinct disinfection strategies for reducing populations of *Pseudomonas paracarnis* A006 on a fresh-cheese model matrix (FCMM) and assessed their effects on lipid integrity. Gaseous ozone and ozonated mist were applied at different concentrations for up to 90 min at 25 °C. Ozone treatments reduced *Pseudomonas* populations by up to 1.20 log CFU/g (gaseous ozone) and 1.67 log CFU/g (ozonated mist), without affecting aerobic mesophilic counts. Ozonation significantly improved color parameters associated with blue discoloration, while only minor changes in water activity were observed. Fatty acid profiles remained largely unaffected under the most intense treatments. This study provides novel evidence on the application of ozonated mist to mitigate *P. paracarnis*-associated blue discoloration in a high-moisture FCMM while preserving lipid integrity, highlighting ozone as a promising and sustainable intervention for fresh-cheese decontamination.

**Supplementary Information:**

The online version contains supplementary material available at 10.1007/s10068-026-02237-2.

## Introduction

Blue discoloration in cheeses is a persistent defect affecting the global dairy industry (Carminati et al., 2019; Carrascosa et al., 2015; Martin et al., 2011; Rodrigues et al., 2021, 2023). This phenomenon, often caused by *Pseudomonas* spp., especially *Pseudomonas fluorescens*, compromises product quality and consumer acceptance (Del Olmo et al., 2018). Beyond modifying sensory properties, these bacteria can raise potential food safety concerns, as *Pseudomonas* biofilms may facilitate the persistence and cross-contamination of pathogenic microorganisms on food-contact surfaces (Pang & Yuk, 2019; Sterniša et al., 2019).

Interest in this issue increased after the widely reported cases of “blue mozzarella” (Nogarol et al., 2013). Blue discoloration has been described in several cheese varieties, including mozzarella (Caputo et al., 2015; Carminati et al., 2019; Sechi et al., 2011), Latin-style fresh cheese (Queso Fresco) (Martin et al., 2011), artisanal cheese from Gran Canaria (Carrascosa et al., 2015), processed cheese (Rodrigues et al., 2023), and Minas Frescal cheese (Rodrigues et al., 2021). In addition to *P. fluorescens,* other species such as *P. carnis, P. paracarnis, P. putida* and *P. lactis* have also been linked to this spoilage defect (Carrascosa et al., 2021; Reichler et al., 2019; Rodrigues et al., 2023, 2024).

Several strategies have been proposed to control contamination by blue pigment-producing *Pseudomonas*. Good Manufacturing Practices (GMP) remain essential, including proper sanitation of equipment, milk pasteurization, and strict control of storage conditions (Law, 2010). In addition to traditional methods, novel approaches have emerged to mitigate the adverse effects of microbial contamination in fresh cheeses. More recently, innovative approaches such as essential oils and their nanoemulsions (Rossi et al., 2025) and pepsin-digested bovine lactoferrin (Caputo et al., 2015) have shown potential against these microorganisms. However, these strategies, as well as other non-thermal technologies such as cold plasma, UV-C irradiation, and aerosol-based systems, may present limitations related to penetration in complex matrices, uniformity of application, and potential effects on product quality (Nemati and Guimarães, 2024; Ricciardi et al., 2020; White et al., 2025). Therefore, further investigation of technologically applicable alternative approaches for dairy systems remains necessary.

Among alternative methods, ozonation has attracted particular attention. Ozone exhibits strong antimicrobial activity and has been shown to control *P. paracarnis* biofilms on stainless steel surfaces commonly used in the food industry (Okolo et al., 2023). Its applications in food matrices, including cheeses, have also been reported (Eramo et al., 2024; Panebianco et al., 2022b; Segat et al., 2014). Ozone can be applied for food decontamination in gaseous, aqueous (ozonated water), or mist forms. The mist form consists of fine droplets of ozonated water suspended in air, combining features of both gas and liquid phases. It maintains good diffusibility and surface coverage typical of gaseous ozone while minimizing the degradation losses and limited stability associated with the aqueous form (Schroer et al., 2022).

In this context, the present study provides novel evidence on the application of ozonated mist for controlling *P. paracarnis*-induced blue discoloration in a fresh-cheese model matrix (FCMM), while simultaneously evaluating its impact on key physicochemical properties, including lipid stability. This approach contributes to advancing the understanding of ozone-based technologies as practical and sustainable interventions for dairy systems.

The effectiveness of ozone derives from its high oxidizing power, which damages cellular components and rapidly inactivates microorganisms (Rangel et al., 2022). Because ozone decomposes into oxygen, it leaves no chemical residues, making it a sustainable and appealing technology for the dairy industry (Botondi et al., 2023). However, it should be noted that gaseous ozone is a greenhouse gas and may pose occupational health risks, including cardiopulmonary diseases (Chen et al., 2023). Therefore, ozone applications should be carried out in closed or well-ventilated systems with proper monitoring to ensure operator safety and environmental compliance.

Despite the increasing number of studies exploring ozone technologies for microbial control, most available data have primarily focused on surface decontamination, biofilm removal on abiotic materials, and the application of ozone in its gaseous form or ozonated water. The use of ozone in mist form remains poorly investigated, particularly regarding its interaction with high-moisture dairy systems and its effectiveness against pigment-producing *Pseudomonas* spp. associated with cheese spoilage. Therefore, a critical gap remains in understanding how ozonated mist behaves as a disinfection strategy in complex food matrices such as fresh cheese, where factors such as water activity, surface heterogeneity, and product composition may influence antimicrobial efficacy.

Because gaseous ozone and ozonated mist differ in their physicochemical behavior, particularly in how relative humidity and aerosol formation influence ozone stability and reactivity, these technologies were treated as distinct disinfection strategies rather than directly comparable delivery methods. Therefore, this study aims to evaluate gaseous ozone and ozonated mist as two distinct disinfection strategies for reducing populations of blue pigment-producing *Pseudomonas* spp. in a FMCC, while also assessing their potential impact on its physicochemical integrity, particularly its lipid fraction. To our knowledge, this is the first study to investigate ozonated mist for controlling *P. paracarnis*-induced blue discoloration in a FMCC, combining microbiological, colorimetric, water activity and lipid profile analyses.

## Materials and methods

### Microorganisms and culture conditions

*P. paracarnis* A006, a blue pigment-producing strain, was the target microorganism used in this study. It was originally isolated from blue Minas Frescal cheese (Rodrigues et al., 2021) and stored at − 80 °C in Brain Heart Infusion (BHI) broth with 20% (v/v) glycerol. Prior to each experiment, the stock culture was inoculated (1% (v/v)) into fresh BHI broth and incubated at 25 °C for 24 h. The refreshed culture was then centrifuged at 3000 g for 10 min (Thermo Electron Led GMBH Megafuge 8R, Germany), and the supernatant was discarded. The pellet was resuspended in 9 mL of 0.85% (w/v) sterile saline solution. The washing step was repeated once under the same conditions, and the final pellet was again resuspended in saline solution.

The optical density (OD) of the cell suspension was measured at 600 nm using a spectrophotometer (Biospectro SP-22, Brazil). The OD600 was adjusted to a range of 0.200–0.280, corresponding to approximately 10^8^ CFU/mL, as estimated from the calibration curve (CFU/mL = 1.77 × 10^9^ × OD600 − 1.53 × 10^8^, R^2^ = 0.972). To confirm the cell concentration, the standardized suspension was serially diluted and plated using the microdrop method (Morton, 2001) on Plate Count Agar (PCA), followed by incubation at 25 °C for 24–48 h. The bacterial suspension was subsequently diluted to achieve a final concentration of 10^5^ CFU/mL for FCMM inoculation.

### Preparation of fresh-cheese model matrix (FCMM)

To simulate spoilage by *Pseudomonas*, a FCMM was developed. This matrix was designed to reproduce key characteristics of starter-free fresh cheese, including rennet coagulation, near-neutral pH, high moisture content, and high water activity (higher than 0.99), typically associated with a short shelf-life (Evert-Arriagada et al., 2012). FCMM production for in situ analyses was based on the method described by Garnier et al. (2018), with adaptations. In brief, pasteurized skim milk was subjected to ultrafiltration using a HFM 180 system (Koch Membrane Systems Inc., USA) equipped with an 80 kDa molecular weight cut-off membrane and a 0.22 µm pore size, at 45 °C. The fat content of the retentate was adjusted to 20 g/kg by adding fresh cream containing 54% fat. Then, sodium chloride (0.7% w/v) was added to the fat-adjusted retentate. The mixture was heat-treated at 95 °C for 2 min under constant stirring.

After heat treatment, the mixture was cooled to 45 °C and rennet (Maxiren XDS, France), previously diluted at a 1:5 ratio (rennet distilled water), was added. Then, 15 mL of the mixture were distributed into sterilized Petri dishes (Ø = 60 mm). The Petri dishes were incubated at 30 °C for 1 h to promote coagulation. After the initial incubation, the dishes were stored at 5 ± 1 °C for 24–48 h, followed by whey drainage.

Subsequently, 200 µL of the standardized bacterial suspension (10^5^ CFU/mL) was inoculated onto the surface of each FCMM. Following inoculation, the plates were gently tilted in different directions to allow the suspension to spread evenly across the entire surface. This procedure ensured a uniform distribution of the bacterial cells over the matrix surface. After inoculation, the initial population of the target bacteria on the FCMM was approximately 10^4^ CFU/g, as a representative intermediate value that allows for measurable growth and response to treatments. The inoculated FCMM were incubated at 5 ± 1 °C for 7 days. This 7-day period was defined based on preliminary assays showing that visible pigment formation occurred only after the fifth day, with no detectable alterations before that point; therefore, intermediate samplings were not considered necessary. FCMM without bacterial inoculum served as negative controls.

### Ozonation

FCMM were subjected to treatments with ozone gas and ozonated mist. Ozone was generated using an M10 I model generator (MyOZONE, Jaguariúna, São Paulo, Brazil), which operated by the corona discharge principle. The generator was supplied with oxygen from a concentrator (EverFlo, Philips Respironics, Murrysville, Pennsylvania, USA) delivering 90% purity.

Ozone gas was generated using the dielectric barrier discharge (DBD) method. In this generation method, oxygen is forced to pass between two electrodes subjected to a high voltage (3–11 kV) (Fang et al., 2008). Between the two electrodes, there is a dielectric layer made of ceramic material and a gap where electrical discharges occur over the oxygen molecules. The high-energy electrons (1–10 eV) generated in the discharge region can dissociate oxygen molecules into atoms, which then collide with oxygen molecules to form ozone (Fang et al., 2008). The generated ozone gas was applied into the treatment chamber through connections installed at the top of the chamber, and the residual ozone was directed to a thermal destructor as shown in Fig. S1 (Supplementary Material).

The treatment chamber, used for both gaseous ozone and ozonated mist applications, measured 0.3 m in width, 0.5 m in height, and 0.4 m in depth, with an internal volume of 0.06 m^3^. Ozone was continuously supplied at a volumetric flow rate of 2 L min⁻^1^. Before each treatment, ozone was circulated within the empty chamber for 30 min to achieve a homogeneous gas distribution. This procedure ensured that, once the samples were introduced, the internal atmosphere contained a uniform and stable ozone concentration representative of the monitored inlet value.

For the generation of ozonized mist, a mist generator model N10 (myOZONE, Jaguariúna, São Paulo, Brazil) was used. This equipment has a water consumption rate of 2 L/h. The mist produced was continuously introduced into the chamber through a transparent polyurethane tube reinforced with copper-coated steel, which was installed at the top of the chamber, as shown in Fig. S2 (Supplementary Material).

The mist was produced through the piezoelectric effect of six ultrasonic transducers immersed in water and positioned inside the mist generator. The generated mist was mixed with ozone gas and directed through the transparent polyurethane tube into the treatment chamber. To induce the mist flow, an axial fan with an inlet and outlet diameter of 0.1 m was used. According to the literature, the size of water droplets may range from 14 to 42 µm (Jian-yong et al., 2011; Zegers et al., 2000).

The ozone gas concentration was determined using the iodometric method, following the procedure described by Rakness et al. (1996) and accepted by the International Ozone Association (IOA). Ozone was bubbled at a known volumetric flow rate through a 2% potassium iodide (KI) solution. This reaction leads to the release of iodine (I_2_), which is then quantified by titration with a sodium thiosulfate (Na_2_S_2_O_3_) solution. To accelerate I_2_ formation, the medium was acidified with 0.5 mol L⁻^1^ sulfuric acid. A 1% starch solution was used as an auxiliary indicator to detect the endpoint.

Based on preliminary assays and previously published data (Panebianco et al., 2022b), two ozone concentrations were selected for the treatments to represent low and high exposure levels within the operational limits of the generator. Two ozone concentrations were evaluated for FCMM treatments. For the gaseous ozone treatment, the lower concentration (GT1) was 0.62 ± 0.18 mg/L, while the higher concentration (GT2) was 5.22 ± 0.55 mg/L, with a gas flow rate of 2.0 L/min. Gaseous ozone was injected into the top of the acrylic chamber, where the FCMM were placed in open Petri dishes. In this way, the gas was distributed within the chamber and surrounded the FCMM throughout the treatment period, ensuring contact with the entire surface.

For the ozonated mist treatment, the input gas concentrations were 9.47 ± 0.28 mg/L (MT1) and 30.27 ± 0.91 mg/L (MT2). Minor variations in ozone concentration are inherent to gas generation systems and were monitored by titration to ensure consistency across treatments. The experiments were conducted at a temperature of 25 °C. In the experiments with ozone gas application, the relative humidity (RH) inside the treatment chamber was 30%. For the experiments with ozonated mist application, the RH was 100%, with water condensation visible on the internal walls of the treatment chamber.

Both treatments were applied in an acrylic chamber, as illustrated in Figs. S1 and S2 (Supplementary Material), respectively. FCMM were placed inside the chamber for ozonation and exposed to ozone for 30, 60, and 90 min. The ozonated mist was conveyed to the chamber through a transparent polyurethane hose reinforced with copper wire. All treatments (30, 60, and 90 min) with ozone gas and ozonated mist were continuously applied throughout the entire exposure period. During the experiments, the ozone injection was constantly monitored to ensure the gas was effectively delivered, avoiding any interference with the treatments. Each treatment was performed in duplicate for each exposure period. Samples, either inoculated or non-inoculated, that were not exposed to ozonation served as controls and were stored under refrigeration (5 °C) until the end of the ozonation treatments.

After each treatment, the chamber was cleaned and disinfected to prevent cross-contamination. All internal surfaces were wiped with paper towels moistened with 70% ethanol to ensure the removal of residues and inactivation of any remaining microorganisms. After the ozonation period, the samples were removed from the chamber. Part of the samples was immediately subjected to microbiological analysis and Aw measurement. The remaining samples were stored at 5 ± 1 °C for 7 days to assess microbial contamination and color changes over time. This experiment was performed in three independent replicates.

### Microbiological analysis of FCMM

For microbiological analysis, approximately 10 g were randomly taken from FCMM weighing about 15 g and contained in the Petri dish. The subsample was weighed and homogenized in 90 mL of sodium citrate solution (20 g/L, pH 7.5) for 2 min using a stomacher-type homogenizer (BagMixer 400, Interscience, France). Serial dilutions were prepared using 0.85% (w/v) sterile peptone saline solution as the diluent (Rodrigues et al., 2021).

For the enumeration of *Pseudomonas* spp., aliquots of the diluted samples were plated using the microdrop method (Morton, 2001) on *Pseudomonas* base agar (PBA) supplemented with cetrimide, fucidin, and cephalosporin (CFC, Oxoid Limited, Thermo Fisher Scientific Inc., UK). The Petri dishes were incubated for 24 h at 25 °C. The PBA was prepared with the following composition: 16 g/L gelatin peptone, 10 g/L tryptone, 10 g/L potassium sulfate, 14 g/L magnesium chloride hexahydrate, 10 g/L lactose, 11 g/L agar, 0.02 g/L bromothymol blue, and pH 7.2 (Machado et al., 2015).

For aerobic mesophilic counts, samples were plated using the pour plate method on PCA and incubated at 36 ± 1 °C for 48 h (Ryser, 2015). Microbial counts were expressed as Log_10_ CFU/g. Reduction in microbial populations was calculated by subtracting the log counts of treated samples from those of the inoculated non-ozonated controls (positive control).

### Instrumental color evaluation and water activity (Aw) measurement of FCMM

Color measurements were conducted after 7 days of refrigerated storage using the CIELAB color system, which provides the color coordinates L*, a*, and b*. Measurements were performed with a colorimeter (Minolta CR 400, Minolta Corporation, Osaka, Japan). Based on the L*, a*, and b* values, chroma or saturation index (C*), hue angle (h*), and total color difference (ΔE*) were calculated according to Francis (1975), Little (1975), and McLellan et al. (1995), using the following Eqs. 1, 2 and 3, respectively.

1 $$C^{*} { } = \sqrt {\left( {a^{*} } \right)^{2} + \left( {b^{*} } \right)^{2} } { }$$

2 $$h^{*} = {\text{ arctg }}\left( {\frac{{b^{*} }}{{a^{*} }}} \right)$$

3 $$\Delta E^{*} = \sqrt {\left( {L^{*} - L_{0}^{*} } \right)^{2 } + \left( {a^{*} - a_{0}^{*} } \right)^{2 } + \left( {b^{*} - b_{0}^{*} } \right)^{2 } } )$$

where L_0_*, a_0_* and b_0_* refer to the color coordinates of non-inoculated and untreated control samples (negative control).

For photographic documentation, the Photographic Register High Quality (PRHQ) protocol developed by Rodrigues et al. (2022) was used to capture standardized images of the samples in Petri dishes. A black cardboard tube was constructed to match the plate dimensions, with a transparent border allowing controlled light entry while minimizing external reflections. A ring-shaped LED light was positioned to provide uniform illumination, and a smartphone (iPhone 13) was mounted above the setup using a cardboard support to ensure consistent image capture. Images were later processed using PowerPoint software to crop, enhance contrast, and remove visual artifacts for optimized visualization.

Portions of FCMM samples were carefully removed from the Petri dishes with a sterile stainless-steel spatula and used to cover the bottom of the sample holder, ensuring full coverage. This approach allowed measurement of the Aw of the FCMM, rather than only the surface layer. Each sample was analyzed at room temperature (25 ± 1 °C). Measurements were performed immediately after ozonation using an AquaLab instrument (model 3 TE, Decagon Devices, USA), which determines Aw by assessing the sample’s hygroscopic equilibrium.

### Lipid profile analysis of ozonated FCMM

To evaluate the impact of ozonation on the fatty acid profile, non-inoculated samples were treated with the highest concentrations of gaseous ozone (GT2: 5.22 ± 0.55 mg/L) and ozonated mist (MT2: 30.27 ± 0.91 mg/L) for 90 min, the longest exposure time in this study. Lipid extraction was performed according to the method of Bligh and Dyer (1959). Briefly, 10 g of FCMM were mixed with methanol, distilled water, and chloroform for three consecutive extractions. The chloroform phase was collected and evaporated at 70 °C using a laboratory hot plate (TECNAL, TE-085, Brazil).

The extracted lipids were transferred to a 7 mL vial, and 1–2 mL of 2% sulfuric acid in methanol was added. The vials were sealed and incubated at 90 °C for 90 min, with shaking at 200 rpm. After cooling, 1 mL of deionized water and 2 mL of hexane were added, followed by vortexing and centrifugation. The hexane phase containing the fatty acid methyl esters (FAMEs) was collected.

FAME derivatization followed the protocols described by Guihéneuf et al. (2001) and Ichihara and Fukubayashi (2010). FAMEs were analyzed using a gas chromatograph (Shimadzu, GC-2010, Japan) equipped with an SP-2560 capillary column. The oven temperature was initially set at 100 °C for 5 min, then ramped to 250 °C at a rate of 5 °C/min, with a final hold at 250 °C for 5 min. Nitrogen was used as the carrier gas, and the Flame Ionization Detector (FID) was maintained at 270 °C.

### Experimental design

The study was conducted in two stages, as shown in Fig. S3 (Supplementary Material). In the first stage, 70 FCMM units were produced and exposed to either gaseous ozone or ozonated mist. Ten units served as controls: five non-inoculated and untreated (negative control) and five inoculated but untreated (positive control). The remaining 60 units received ozone treatments: 30 units with gaseous ozone and 30 units with ozonated mist, each at two concentrations and three exposure times (30, 60, and 90 min). For each of the six concentration–time combinations, five units were tested. Of these, three were analyzed immediately after treatment (Day 0), and two were stored under refrigeration for 7 days (Day 7) to assess blue pigment production.

At time 0, one unit was used for Aw measurement, while the remaining two were subjected to microbiological analysis. After 7 days of storage, the two cold-stored units were evaluated for color (non-destructively) and microbial contamination. The entire experiment was performed in triplicate, resulting in a total of 210 FCMM units.

In the second stage, nine additional FCMM units were produced to evaluate their fatty acid profile. Three units were treated with the highest ozone concentration in gaseous form (GT2), three with the highest ozone concentration in mist form (MT2), and three were kept untreated as controls. For both ozone treatments, exposure time was 90 min.

### Statistical analysis

Statistical analyses and data visualization were performed using R software version 4.3.3 and RStudio 2023.03.0 + 386 (R Core Team, 2023; RStudio Team, 2020). The packages ‘ExpDes.pt’, ‘conover.test’, ‘stats’, and ‘ggplot2’ were used to perform the analyses (Dinno, 2017; Ferreira et al., 2013; R Core Team, 2023; Wickham, 2016).

Data normality and homogeneity of variances were assessed using the Shapiro–Wilk and Bartlett tests, respectively. When these assumptions were not met (p < 0.05), indicating deviation from parametric test requirements, non-parametric tests (Kruskal–Wallis) were applied.

The reduction in *Pseudomonas* and mesophilic populations, as well as the color parameters lightness (L*), hue angle (h*), and total color difference (ΔE*), were analyzed using the Kruskal–Wallis test, followed by Conover’s post-hoc test. Water activity (Aw) data were analyzed using analysis of variance (ANOVA) in a completely randomized design with a 2 × 4 factorial arrangement, comprising two ozone concentrations and four exposure times. When significant, treatment means were compared using Tukey’s test. Fatty acid data from ozonated and non-ozonated samples were compared using Student’s *t-*test. In addition to *p*-values, effect sizes were considered to support the interpretation of treatment effects. A significance level of *p* ≤ 0.05 was adopted for all statistical tests.

## Results and discussion

### Control of microbiological contamination in FCMM

Gaseous ozone and ozonated mist were evaluated as strategies to reduce microbial contamination and blue discoloration in a FCMM. The populations of aerobic mesophilic bacteria and *Pseudomonas* spp. were assessed immediately after ozonation (0 days) and after 7 days of refrigerated storage.

No significant differences (*p* > 0.05) were observed in the reduction of mesophilic populations (log CFU/g) among samples treated with different concentrations of gaseous ozone at the evaluated exposure times (30, 60, and 90 min), immediately after treatment or after storage (Table 1). However, for the ozonated mist, significant differences were observed between different concentrations on day 7 within each exposure time, and between storage periods (0 and 7 days) for MT1 at each exposure time (Table 1).

**Table 1:** Reduction of mesophilic aerobic populations (log CFU/g) in fresh-cheese model matrix treated with gaseous ozone (GT1 and GT2) and ozonated mist (MT1 and MT2) for 30, 60, and 90 min

Treatments	Exposure time		
	30 min	60 min	90 min
Gas			
Day 0			
GT1	0.67 ± 0.15	0.73 ± 0.15	0.83 ± 0.25
GT2	0.90 ± 0.20	1.07 ± 0.31	1.20 ± 0.20
Day 7			
GT1	0.80 ± 1.04	0.87 ± 0.99	0.83 ± 0.84
GT2	0.90 ± 0.44	0.97 ± 0.40	1.47 ± 0.12
Mist			
Day 0			
MT1	1.00 ± 1.04^X^	1.10 ± 1.04^X^	1.17 ± 1.07^X^
MT2	1.23 ± 1.10	1.27 ± 1.08	1.47 ± 0.90
Day 7			
MT1	0.23 ± 0.06 B^X^	0.30 ± 0.00 B^X^	0.37 ± 0.06 B^X^
MT2	0.97 ± 0.49 A	1.10 ± 0.52 A	1.20 ± 0.61 A

Uppercase letters in the column represent a statistical difference (*p* ≤ 0.05) between the treatments within each exposure time. An (^X^) indicates a statistical difference between the storage days (0 and 7) within each treatment at each exposure time

*GT1* gaseous ozone with an input concentration of 0.62 ± 0.18 mg/L, *GT2* gaseous ozone with a concentration of 5.22 ± 0.55 mg/L, *MT1* mist with an input ozone concentration of 9.47 ± 0.28 mg/L, *MT2* mist with an input ozone concentration of 30.27 ± 0.91 mg/L

Nevertheless, it is important to highlight that, despite these statistical differences, the 90-min exposure treatment produced reductions of about 1.5 log CFU/g from an initial mesophilic load of 4 log CFU/g. Although a residual population remained, such a reduction may be meaningful within the preventive context of this study, as the treatment substantially lowered the microbial load and limited bacterial proliferation during storage. The original mesophilic bacterial counts (log CFU/g) for the control and all treatments are provided in Supplementary Material—Table S1 to illustrate the absolute microbial levels over storage.

The greatest microbial reduction under the evaluated conditions was observed in samples treated with ozonated mist at the highest concentration (MT2). After 90 min of exposure, the reduction reached 1.47 ± 0.90 log CFU/g (approximately 96.61%) on day 0, and 1.20 ± 0.61 log CFU/g (approximately 93.69%) on day 7 (Table 1).

The effect of gaseous ozone and ozonated mist on the reduction of *Pseudomonas* spp. counts varied according to the treatment parameters (Table 2). *Pseudomonas* spp. was used here to reflect that, although FCMM were inoculated with *P. paracarnis* A006, microbial enumeration was performed on PBA agar supplemented with CFC, which allows the growth of other *Pseudomonas* species that may be naturally present in the samples. At the lowest gas concentration (GT1), exposure for 90 min resulted in a modest reduction of 0.20 ± 0.17 log CFU/g (36.90%). However, this reduction was not statistically significant compared with the shorter exposure times within GT1 (*p* > 0.05), although differences between storage days (0 and 7) were significant (^X^). In contrast, treatment with the highest gas concentration (GT2) led to a significantly greater reduction of 1.20 ± 0.20 log CFU/g (93.96%) after the same exposure time. Untreated FCMM inoculated with an initial *Pseudomonas* population of approximately 10^4^ CFU/g reached approximately 10^8^ CFU/g after 7 days of storage at 5 °C, reinforcing the need for effective microbial control strategies. The original *Pseudomonas* counts (log CFU/g) for the control and all treatments are provided in Supplementary Table S2, illustrating the absolute microbial levels over storage.

**Table 2:** Reduction of *Pseudomonas* spp. counts (log CFU/g) in fresh-cheese model matrix treated with gaseous ozone (GT1 and GT2) and ozonated mist (MT1 and MT2) for 30, 60, and 90 min

Treatments	Exposure time		
	30 min	60 min	90 min
Gas			
Day 0			
GT1	0.57 ± 0.31^X^	0.57 ± 0.31^X^	0.67 ± 0.31 B^X^
GT2	0.70 ± 0.26^X^	0.73 ± 0.23	1.07 ± 0.06 A
Day 7			
GT1	0.17 ± 0.12^X^	0.17 ± 0.12 B^X^	0.20 ± 0.17 B^X^
GT2	0.30 ± 0.17 b^X^	0.47 ± 0.15 bA	1.20 ± 0.20 aA
Mist			
Day 0			
MT1	0.43 ± 0.12	0.43 ± 0.12 B	0.50 ± 0.00 B
MT2	0.67 ± 0.15	0.80 ± 0.26 A^X^	0.97 ± 0.06 A
Day 7			
MT1	0.17 ± 0.12 bB	0.33 ± 0.25 abB	0.60 ± 0.36 aB
MT2	0.87 ± 0.32 bA	1.37 ± 0.23 abA^X^	1.67 ± 0.42 aA

Lowercase letters differing within the same row represent a statistical difference (*p* ≤ 0.05) between the exposure times within each treatment. Uppercase letters differing in the column represent a statistical difference between the treatments within each exposure time. An (^X^) indicates a statistical difference between the storage days (0 and 7) within each treatment at each exposure time

*GT1* gaseous ozone with an input concentration of 0.62 ± 0.18 mg/L, *GT2* gaseous ozone with a concentration of 5.22 ± 0.55 mg/L, *MT1* mist with an input ozone concentration of 9.47 ± 0.28 mg/L, *MT2* mist with an input ozone concentration of 30.27 ± 0.91 mg/L

Immediately after ozonation (Day 0), reductions ranging from 0.67 to 1.47 log CFU/g were observed, depending on the treatment. After 7 days of refrigerated storage, populations increased in all treatments, although the final counts remained lower in samples exposed to the highest ozone concentrations (GT2 and MT2) (Table S2), indicating a delayed regrowth compared to the control. At the highest concentration (MT2) and the longest exposure time (90 min), a reduction of 1.67 ± 0.42 log CFU/g (97.86%) was achieved. Although ozonated mist resulted in higher microbial reductions under the conditions tested, it is important to emphasize that gaseous ozone and ozonated mist were applied under different relative humidity and ozone concentration conditions. Therefore, the observed differences should be interpreted with caution, as they reflect condition-specific outcomes rather than a direct comparison of the intrinsic efficacy of each treatment (Tables 2 and S2). Although these reductions were moderate, the results suggest that ozonated mist can reduce microbial loads under the specific conditions tested, particularly at higher ozone concentrations and longer exposure times.

No significant reduction in the aerobic mesophilic population was observed in FCMM treated with ozone gas (Table 1), consistent with the findings of Panebianco et al. (2022b), who observed no significant differences in aerobic bacterial counts between ozone-treated and untreated dairy products. It is important to note that aerobic mesophiles represent a broad and heterogeneous group of bacteria, including multiple genera that can grow under moderate temperatures, whereas *Pseudomonas* spp. is a specific genus commonly associated with spoilage, blue pigmentation, and proteolytic activity in cheeses (Perin et al., 2017). Therefore, while ozone treatment effectively reduced *Pseudomonas* spp. counts, this does not necessarily translate to a reduction in total aerobic mesophilic counts, as these encompass many other bacterial populations naturally present in the FCMM, including non-spoilage microorganisms. This taxonomic diversity may explain the limited impact of gaseous ozone on mesophilic counts. Moreover, the spatial distribution of microorganisms within the FCMM may have influenced the outcomes. While *P. paracarnis* A006 was specifically inoculated on the surface, where ozone exerts its most pronounced effects, the mesophilic microbiota is likely more uniformly distributed throughout the matrix, potentially limiting the efficacy of surface-directed ozonation (Da Silva et al., 2024).

The antimicrobial efficacy of ozone can vary considerably across bacterial species and even among strains, as demonstrated by Von Gunten (2003). This variability is influenced by several intrinsic factors, including cell wall structure, physiological state, and the effectiveness of cellular repair mechanisms (Patil et al., 2011). Furthermore, ozone application parameters, such as ozone concentration and exposure time, play a critical role in determining the bactericidal efficiency of ozonation.

In the present study, both ozone gas and ozonated mist significantly reduced *Pseudomonas* populations, with efficacy directly influenced by ozone concentration, exposure, and storage time (Table 2). Cabral et al. (2024) also highlighted ozonated mist as a promising alternative for microbial inactivation. Additionally, combining ozonated mist with other disinfection methods may enhance antimicrobial effects through synergistic interactions, as reported by Epelle et al. (2023).

In this study, it is essential to distinguish between gaseous ozone and ozonated mist, as these systems differ fundamentally in terms of phase distribution, mass transfer, and physicochemical behavior. In gaseous ozone applications, relative humidity (RH) plays a critical role in determining ozone efficacy. Experimental evidence indicates that ozone exhibits markedly higher antimicrobial activity under humid conditions, whereas substantially greater ozone exposures are required to achieve comparable microbial inactivation at low RH (Elford and van den Ende, 1942; McClurkin et al., 2013; Ozkan et al*.,* 2011). In contrast, ozonated mist constitutes a heterogeneous gas–liquid system composed of dispersed microdroplets, which have been reported to range from 14 to 42 μm in diameter (Zegers et al., 2000), in which ozone partitions among the gas phase, the droplet interface, and the aqueous microenvironment (Da Silva et al. 2024). Within these microdroplets, localized aqueous-phase processes may occur, including ozone decomposition and formation of reactive oxygen species (ROS). However, unlike ozonated water, these processes are spatially constrained and governed by mass transfer limitations and interfacial phenomena.

Regardless of the application mode, both molecular ozone and ROS generated in mist systems play a key role in microbial inactivation through oxidative interactions with organic constituents of microbial cells and biofilm matrices. In the conditions evaluated in this study, molecular ozone can directly react with cell envelope components, including lipids, proteins, and polysaccharides, leading to structural damage and loss of cellular integrity (Khadre et al., 2001). In mist systems, short-lived ROS formed within microdroplets may further enhance this effect through highly localized, non-selective oxidation. In both cases, these oxidative processes also target the extracellular polymeric substances that constitute biofilms (Santos et al., 2025), promoting partial disruption of their structure and thereby enhancing microbial susceptibility to oxidative stress. Nevertheless, ozone primarily acts at the surface where microorganisms are exposed, and its penetration into deeper layers is limited. This surface-oriented activity may partially disrupt microbial aggregates or biofilm-like structures. However, overall reductions tend to remain moderate due to incomplete penetration and heterogeneous microbial distribution within the matrix (Megahed et al., 2018; Panebianco et al., 2022a; Patil et al., 2011).

Nevertheless, both approaches exhibited a consistent trend: the highest ozone concentration and the longest exposure time resulted in the greatest microbial reductions, with decreases of 1.20 ± 0.20 log CFU/g (93.69%) for gaseous ozone (GT2), and 1.67 ± 0.42 log CFU/g (97.86%) for ozonated mist (MT2) (Table 2). Although these reductions are moderate, they are relevant in the context of post-processing contamination in high-moisture cheeses. In this scenario, reducing the initial population can play a critical role in delaying microbial growth and extending product shelf life. Importantly, the reductions observed in this study are lower than those commonly reported in studies aiming at higher levels of microbial inactivation. This is consistent with previous studies showing that ozone-based treatments, particularly in dairy matrices, often result in limited microbial reductions depending on application conditions (Botondi et al., 2023; Torlak and Sert, 2013). Therefore, ozone treatments should not be considered as standalone decontamination or sterilization methods, but rather as complementary or preventive strategies aimed at mitigating spoilage and controlling post-pasteurization contamination.

### Water activity (Aw)

Aw of the FCMM was measured immediately after ozonation to evaluate whether the treatments induced changes in this physicochemical parameter. Analysis of variance for the two evaluated factors (ozone concentration and exposure time) showed no significant interaction between them (gas: *p* = 0.2434; mist: *p* = 0.1601). Thus, the main effects of each factor were analyzed separately.

**Fig. 1:**
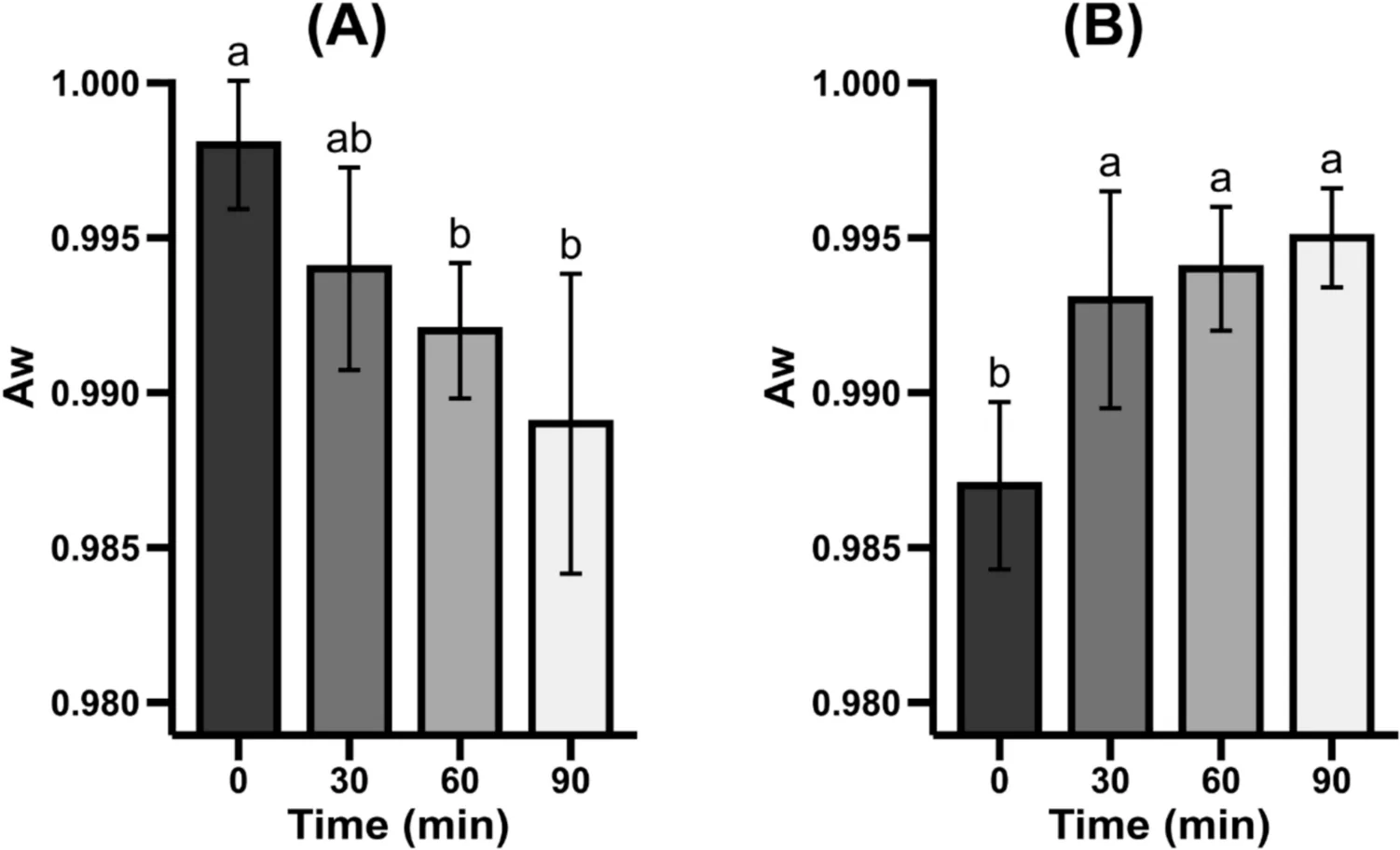
Water activity (Aw) of fresh-cheese model matrix treated with gaseous ozone (**A**) and ozonated mist (**B**), including untreated controls (0 min) and samples exposed for 30, 60, and 90 min. Distinct lowercase letters indicate a statistical difference at the 5% significance level

In the gas treatment, a significant difference was observed between concentrations (*p* = 0.004), with GT1 showing a higher Aw (0.995 ± 0.003) compared to GT2 (0.991 ± 0.005). Similarly, in the ozonated mist treatment, Aw also differed significantly between concentrations (*p* = 0.0071), with MT2 exhibiting higher values (0.994 ± 0.004) than MT1 (0.991 ± 0.003). For the exposure time, Aw decreased over time with gaseous ozone (Fig. 1A), while ozonated mist increased Aw after 30 min (Fig. 1B).

The treatments also influenced the Aw of the FCMM. Gas ozonation significantly reduced Aw with increasing exposure time (Fig. 1), likely due to the low RH (30%) of ozone gas (Cuong et al., 2019), which is generated from oxygen. This reduction in RH may contribute to moisture loss from the FCMM surface, thereby decreasing Aw during treatment. In contrast, FCMM treated with ozonated mist exhibited an increase in Aw, indicating moisture absorption from the mist (Fig. 1). This effect is likely due to the water content of the mist and its initial interaction with the product surface. Segat et al. (2014) similarly reported that ozone can affect the RH in cheeses.

The growth of *Pseudomonas* spp. is closely associated with Aw, as these bacteria can proliferate in environments with Aw values above 0.97 (Dimassi et al., 2020). Although variations in Aw were observed across treatments, none of the samples presented Aw values below this threshold. Therefore, the reduction in Aw induced by ozone gas, even under prolonged exposure (Fig. 1), was not sufficient to inhibit *P. paracarnis* growth on its own. These results suggest that the microbial inhibition was primarily due to the oxidative effect of ozone, rather than to alterations in water availability, reinforcing the antimicrobial potential of the treatment.

Although mist ozonation caused a statistically significant increase in water activity, the values remained within the typical range reported for FCMM (0.991–0.995). From a microbiological standpoint, such small variations are unlikely to compromise product safety or stability during refrigerated storage. Moreover, ozone exerts broad-spectrum antimicrobial activity, which includes the ability to inactivate both spoilage and pathogenic microorganisms (Bigi et al., 2021; Gibson et al., 2019). Therefore, even if a slight increase in Aw occurs, the concurrent antimicrobial action of ozone is expected to outweigh any potential impact on microbial proliferation. Nonetheless, these findings highlight the importance of monitoring physicochemical parameters when applying ozone-based treatments to high-moisture dairy matrices.

### Colorimetric analysis of FCMM

**Fig. 2:**
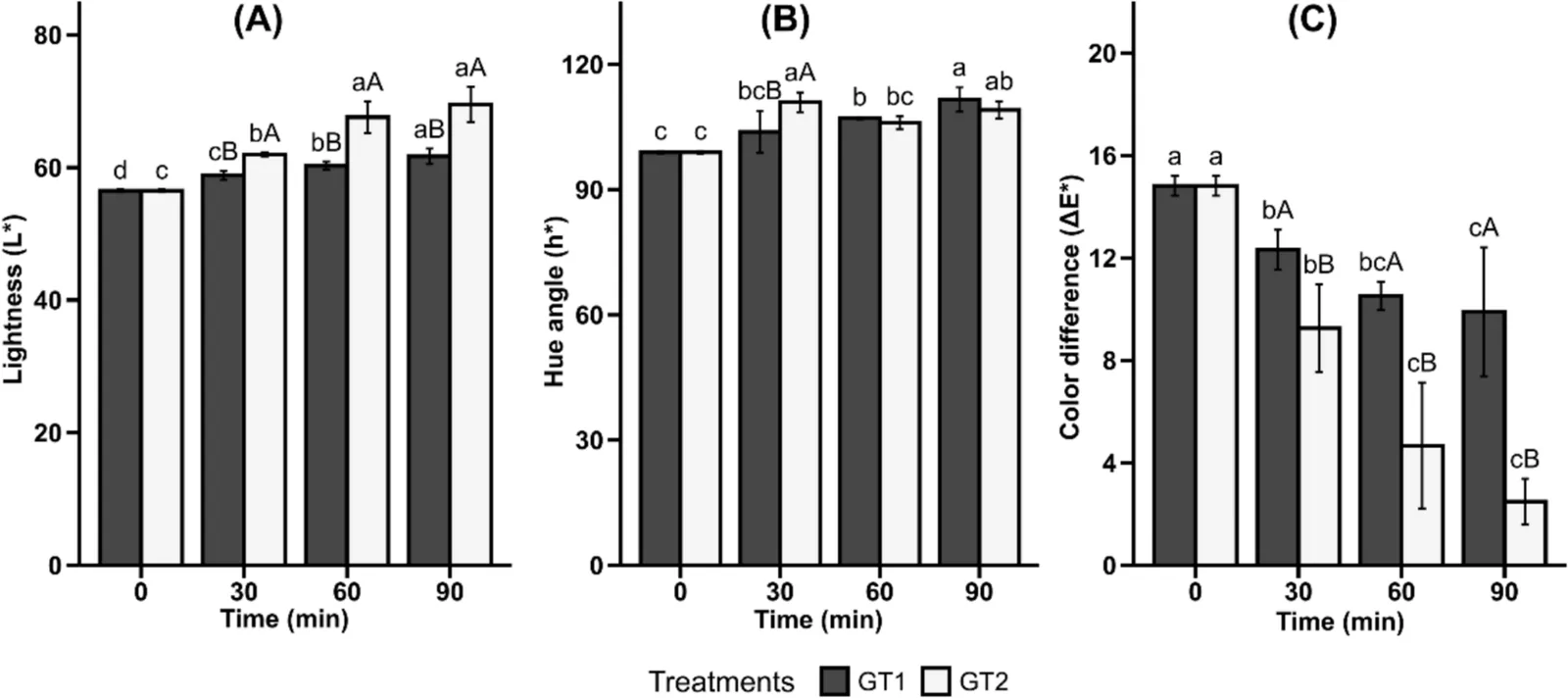
Mean variation in lightness (L*) (**A**), color hue (h*) (**B**), and color difference (ΔE*) (**C**) in fresh-cheese model matrix treated with two concentrations of gaseous ozone (GT1 and GT2) over different exposure times, after 7 days of storage. GT1: gaseous ozone with an input concentration of 0.62 ± 0.18 mg/L; GT2: gaseous ozone with a concentration of 5.22 ± 0.55 mg/L. Different lowercase letters indicate significant differences (*p* ≤ 0.05) between exposure times within each treatment. Different uppercase letters indicate significant differences between treatments within each exposure time

**Fig. 3:**
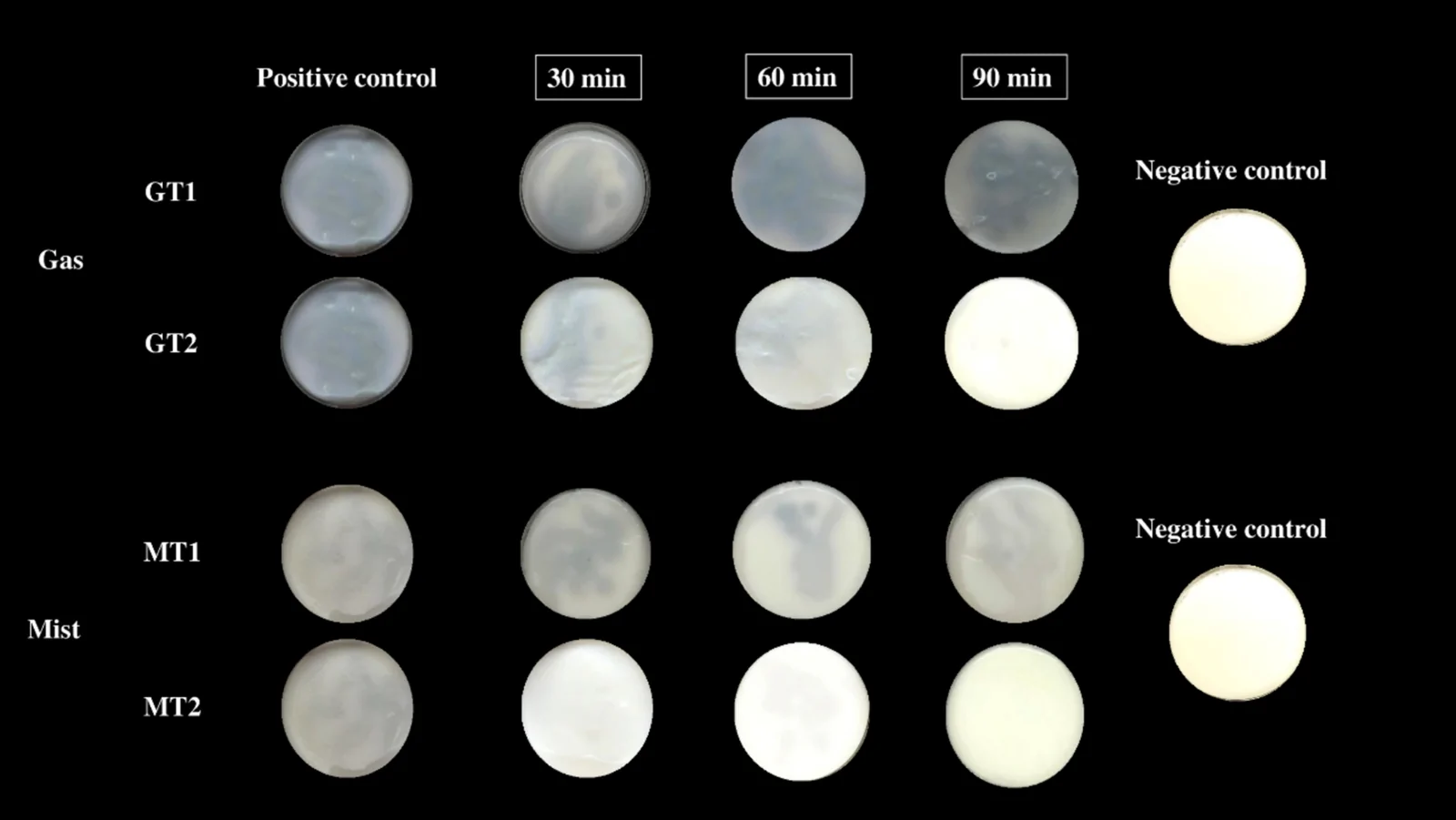
Photographic records of fresh-cheese model matrices under the different experimental conditions: non-inoculated and untreated cheeses (negative control), inoculated with *P. paracarnis* A006 but untreated samples (positive control), and samples inoculated with *P. paracarnis* A006 and treated with gaseous ozone (GT1 and GT2) or ozonated mist (MT1 and MT2) for 30, 60, and 90 min

**Fig. 4:**
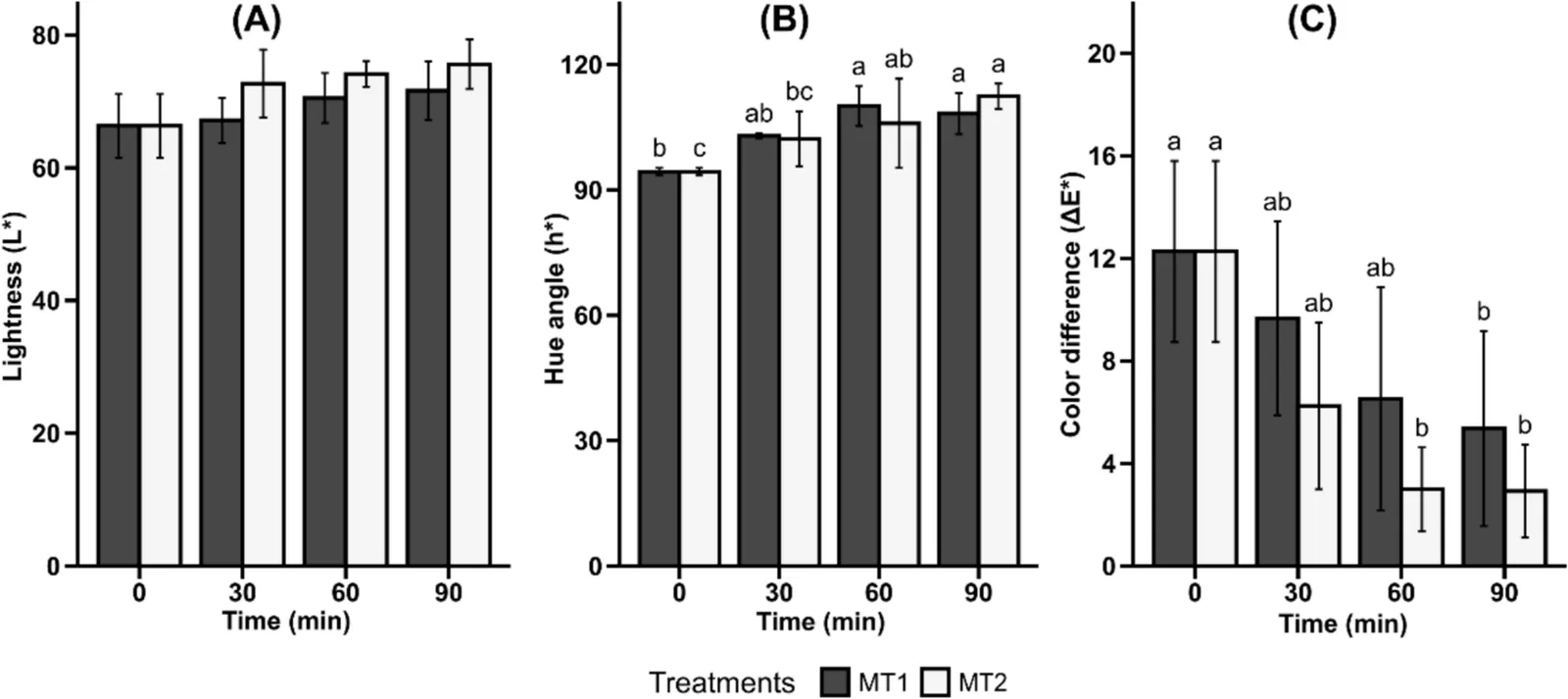
Mean variation in lightness (L*) (**A**), hue angle (h*) (**B**), and color difference (ΔE*) (**C**) of fresh-cheese model matrix treated with two ozone mist concentrations (MT1 and MT2) across different exposure times, after 7 days of storage. Lowercase letters indicate a significant difference (*p* ≤ 0.05) between exposure times within each treatment

Colorimetric analysis of FCMM treated with gaseous ozone after 7 days of cold storage provided insights into the effects of ozone concentration and exposure time on its color, particularly regarding the reduction of blue discoloration (Figs. 2, 3, and 4). No significant differences were observed in the chroma (C*) values for samples ozonized either with gas or mist. However, significant effects were found for lightness (L*), hue angle (h*), and total color difference (ΔE*). The mean values for these parameters are presented in Tables S3 and S4 (Supplementary Material).

Treatment with gaseous ozone led to an increase in lightness (L*) compared to the inoculated and untreated control (positive control). Longer exposure times were more effective in enhancing FCMM lightness at both tested concentrations (Fig. 2A). Samples treated with the higher ozone concentration (GT2) exhibited significantly greater lightness than those treated with the lower concentration (GT1) (Table S3, Fig. 2) across all exposure times (30, 60, and 90 min). FCMM treated with the highest concentration GT2 for 90 min exhibited the lowest ΔE* value (2.49 ± 0.89) (Figs. 2 and 3), indicating the smallest color change among the evaluated gaseous ozone conditions.

An increase in hue angle (h*) was also observed with increasing exposure time. This indicates an angular shift opposite to the blue region, corresponding to the gradual disappearance of the bluish tone, which was consistent with the reduction in the population of blue pigment-producing *Pseudomonas* spp. following ozone treatment (Figs. 2 and 3). Additionally, a reduction in the total color difference (ΔE*) was observed as exposure time increased, indicating that the color of contaminated samples became more similar to the non-inoculated control (negative control) following ozone treatment (Figs. 2 and 3). The corresponding ΔE* values for each treatment and exposure time are presented in the supplementary material (Table S3).

Colorimetric analysis of FCMM treated with ozonated mist after 7 days of storage also highlighted the influence of exposure time on color parameters, particularly in reducing the blue discoloration (Fig. 4). For both evaluated ozone mist concentrations (MT1 and MT2), no statistically significant differences in lightness (L*) (*p* = 0.1269) were observed. However, a significant increase in hue angle (h*) was noted with longer exposure time, accompanied by a corresponding decrease in total color difference (ΔE*), indicating progressive reduction of discoloration (Fig. 4). Photographic records (Fig. 3) further illustrate that treatments using the highest mist concentration (MT2) were more effective in mitigating the FCMM spoilage.

Colorimetric analysis of ozone-treated FCMM after 7 days of storage provided crucial insights into the effects of ozone concentration and exposure time on its appearance, particularly in the reduction of blue pigment production, as shown in Fig. 3. Cheese color is a key sensory attribute that strongly influences consumer acceptance and perception of product quality (Tabla & Roa, 2022). In this study, treatments using the highest concentrations of gaseous ozone (GT2) and ozonated mist (MT2) were the most effective in mitigating blue discoloration. Appropriate adjustment of ozonation parameters can contribute to the preservation of FCMM visual quality.

Beyond microbial inactivation, ozonation also influenced the visual quality of the FCMM. In this study, ozone treatments were applied before blue pigmentation became visible, acting as a preventive approach rather than as a corrective treatment. Although the microbial reductions achieved were lower than 2 log CFU/g, these treatments had a notable impact on the colorimetric properties of the FCMM. The attenuation or absence of pigmentation may be associated with both the reduction in viable *Pseudomonas* populations and possible physiological stress imposed by ozone exposure. Although sublethal injury has been reported previously (Pyatkovskyy et al., 2025), this mechanism was not directly assessed here. Therefore, the delayed pigment formation observed is more likely explained by the lower initial microbial loads and the altered growth dynamics of the surviving cells during storage.

### Fatty acid profile

To assess the impact of ozone treatment on the fatty acid profile, only the most effective conditions (highest ozone concentrations GT2 and MT2) and the longest exposure time (90 min) were selected, based on prior results related to *Pseudomonas* spp. reduction and color improvement. Fatty acid distribution was determined for each sample to evaluate potential changes associated with ozone treatments.

The composition of FAMEs identified in the samples is shown in Table S5 (Supplementary Material). A total of nine fatty acid esters were detected in both treated and untreated samples: C8:0 (caprylic), C10:0 (capric), C12:0 (lauric), C14:0 (myristic), C16:0 (palmitic), C16:1 (palmitoleic), C18:0 (stearic), C18:1n9c (oleic), and C18:2n6c (linoleic) (Supplementary Material—Table 5S). Saturated fatty acid esters were predominant in all samples, representing 70.90–72.58% of the total esters, as shown in Table S6 (Supplementary Material).

Ozone treatment did not significantly affect the proportions of saturated, monounsaturated, and unsaturated fatty acids (Supplementary Material—Table S6). However, samples treated with gaseous ozone at the highest concentration (GT2) exhibited a significant increase in polyunsaturated fatty acid esters (*p* = 0.0246), an effect not observed in samples treated with ozonated mist.

Although the oxidative nature of ozone raises concerns about potential lipid degradation, particularly through lipid oxidation processes that may compromise fatty acid integrity, the results of this study indicate a limited impact on the lipid profile of treated FCMM. Significant differences were observed only in the polyunsaturated fatty acid (PUFA) content of FCMM treated with gaseous ozone (GT2), while no alterations were detected in saturated or monounsaturated fatty acids (Supplementary Material—Table S6). These findings suggest that, under the applied conditions, ozonation was effective in controlling microbial and pigment-related spoilage without substantially compromising the chemical stability of lipids.

Nonetheless, the possibility of ozone-induced lipid oxidation should not be disregarded. This process is known to influence the stability, flavor, and texture of dairy products, key factors for consumer acceptance (Eramo et al., 2024; Scudino et al., 2024). Therefore, while ozone presents promising antimicrobial benefits, its application in dairy matrices must be carefully optimized to avoid compromising lipid quality and sensory characteristics.

Ozonation, both as a gas and as ozonated mist, may be considered suitable for food-processing environments when conducted in accordance with established safety guidelines and regulatory standards. Its use in controlled environments proved effective in reducing surface contamination without leaving chemical residues, making it a safe and sustainable alternative for food preservation (Eramo et al., 2025). Importantly, the efficacy of ozone treatment is highly dependent on precise control of application parameters such as concentration, exposure time, and environmental conditions (Leite et al., 2024).

Although the FCMM used in this study was designed to mimic key physicochemical properties of FCMM, it does not fully reproduce the complexity of industrial products, including microbiota, structural heterogeneity, and processing variability. These factors may influence ozone diffusion, microbial tolerance, and overall treatment efficacy. Therefore, the applicability of the results to real industrial conditions should be interpreted with caution. Further studies in industrial environments are necessary to validate the effectiveness, scalability, and practical feasibility of ozone-based interventions under real processing conditions.

This study demonstrated that ozone, applied either as gas or as ozonated mist, reduced *Pseudomonas* contamination and mitigated blue discoloration in a FCMM. The antimicrobial efficacy of ozone was strongly dependent on concentration and exposure time, with both methods showing a reduction in the microbial population. Both treatments had minimal impact on fatty acid composition, indicating that ozone can be applied without compromising key nutritional attributes. These findings support further evaluation of ozone as a sustainable, residue-free technology for microbial control in fresh cheeses, including its application in mist form under appropriate conditions. Further studies under industrial conditions are recommended to validate its large-scale applicability and optimize process parameters.

## Supplementary Information

Below is the link to the electronic supplementary material.

Supplementary file1 (DOCX 904 KB)
