# Supplementary material for: Ozone-based interventions to mitigate Pseudomonas paracarnis-induced blue discoloration in fresh cheese
Source: Food Sci Biotechnol. 2026 Jul 21;35(10):2943–57. doi: 10.1007/s10068-026-02237-2 (PMC13518684; doi:10.1007/s10068-026-02237-2)
Supplement: Supplementary file 1 [file 10068_2026_2237_MOESM1_ESM.docx]

**Supplementary Material**

**Ozone-based interventions to mitigate *Pseudomonas paracarnis*-induced blue discoloration in fresh cheese**

Juniel Marques de Oliveira^a^, Laysha Neto de Paula Campos^b^, Emanoel Henrique Fialho Ferreira^c^, Letícia Elisa Rossi^a^, Rafaela da Silva Rodrigues^d^, Marcus Vinicius de Assis Silva^c^, Ernandes Rodrigues de Alencar^c^, Marcio Arêdes Martins^c^, Mauricio de Oliveira Leite^c^, Antônio Fernandes de Carvalho^b^, Solimar Gonçalves Machado^b,*^

^a^ *Department of Microbiology, Universidade Federal de Viçosa, Viçosa, MG, Brazil*

^b^ *Department of Food Technology, Universidade Federal de Viçosa, Viçosa, MG, Brazil*

^c^ *Department of Agricultural Engineering, Universidade Federal de Viçosa, Viçosa, MG, Brazil*

^d^ *Department of Veterinary Medicine, Universidade Federal de Viçosa, Viçosa, MG, Brazil*

**Email address of each author:** [juniel.oliveira@ufv.br](mailto:juniel.oliveira@ufv.br); [laysha.campos@ufv.br](mailto:laysha.campos@ufv.br); [emanoel.ferreira@ufv.br](mailto:emanoel.ferreira@ufv.br); [leticia.rossi@ufv.br](mailto:leticia.rossi@ufv.br); [rafaela.s.rodrigues@ufv.br](mailto:rafaela.s.rodrigues@ufv.br); [marcus.assis@ufv.br](mailto:marcus.assis@ufv.br); [ernandes.alencar@ufv.br](mailto:ernandes.alencar@ufv.br); [aredes@ufv.br](mailto:aredes@ufv.br); [mauricio.leite@ufv.br](mailto:mauricio.leite@ufv.br); [antoniofernandes@ufv.br](mailto:antoniofernandes@ufv.br); [solimar.machado@ufv.br](mailto:solimar.machado@ufv.br)

*** Corresponding author:** Solimar Gonçalves Machado, Av. Peter Henry Rolfs, Department of Food Technology, s/n - Campus Universitário, Viçosa - MG, Brazil, 36570-900. E-mail: [solimar.machado@ufv.br](mailto:solimar.machado@ufv.br)

**Figure S1**. Experimental setup for exposing fresh-cheese model matrix to gaseous ozone.

**Figure S2**. Experimental setup for exposing fresh-cheese model matrix to ozonated mist.

**Figure S3**. Experimental design of the study. First stage: 70 fresh-cheese model matrix units were assigned to controls or treated with gaseous ozone (GT1, GT2) or ozonated mist (MT1, MT2) at different concentrations and exposure times (30, 60, 90 min), followed by analyses at day 0 (T0) and day 7 (T7). Second stage: nine additional units were used to assess fatty acid profiles under the most effective conditions (GT2 and MT2, 90 min). GT1: 0.62 ± 0.18 mg/L; GT2: 5.22 ± 0.55 mg/L; MT1: 9.47 ± 0.28 mg/L; MT2: 30.27 ± 0.91 mg/L. n: sample size; Positive control: inoculated but untreated samples; Negative control: non-inoculated and untreated samples.

**Table S1** – Mesophilic aerobic populations (log CFU/g) in fresh-cheese model matrix treated with gaseous ozone (GT1 and GT2) and ozonated mist (MT1 and MT2) immediately after ozonation (Day 0) and after 7 days of refrigerated storage (Day 7).

| **Treatments** |  | **Exposure Time** | | |
| --- | --- | --- | --- | --- |
|  | **Control** | **30 min** | **60 min** | **90 min** |
|  | *Gas* | | | |
|  | **Day 0** | | | |
| **GT1** | 4.19 ± 0.45 | 3.56 ± 0.86 | 3.46 ± 0.06 | 3.30 ± 0.41 |
| **GT2** | 4.19 ± 0.45 | 3.30 ± 0.60 | 3.15 ± 0.39 | 3.00 ± 1.45 |
|  | **Day 7** | | | |
| **GT1** | 8.92 ± 0.21 | 8.12 ± 0.14 | 8.05 ± 0.13 | 8.09 ± 0.09 |
| **GT2** | 8.92 ± 0.21 | 8.02 ± 0.18 | 7.95 ± 0.23 | 7.45 ± 0.33 |
|  | *Mist* | | | |
|  | **Day 0** | | | |
| **MT1** | 4.18 ± 0.47 | 3.18 ± 0.28 | 3.08 ± 0.28 | 3.01 ± 0.30 |
| **MT2** | 4.18 ± 0.47 | 2.96 ± 0.35 | 2.92 ± 0.37 | 2.71 ± 0.25 |
|  | **Day 7** | | | |
| **MT1** | 8.89 ± 0.20 | 8.66 ± 0.31 | 8.59 ± 0.17 | 8.52 ± 0.19 |
| **MT2** | 8.89 ± 0.20 | 7.91 ± 0.59 | 7.79 ± 0.60 | 7.70 ± 0.61 |

GT1: gaseous ozone with an input concentration of 0.62 ± 0.18 mg/L; GT2: gaseous ozone with a concentration of 5.22 ± 0.55 mg/L; MT1: mist with an input ozone concentration of 9.47 ± 0.28 mg/L; MT2: mist with an input ozone concentration of 30.27 ± 0.91 mg/L.

**Table S2 –** *Pseudomonas* spp. populations (log CFU/g) in fresh-cheese model matrix treated with gaseous ozone (GT1 and GT2) and ozonated mist (MT1 and MT2) immediately after ozonation (Day 0) and after 7 days of refrigerated storage (Day 7).

| **Treatments** |  | **Exposure Time** | | |
| --- | --- | --- | --- | --- |
|  | **Control** | **30 min** | **60 min** | **90 min** |
|  | *Gas* | | | |
|  | **Day 0** | | | |
| **GT1** | 4.44 ± 0.23 | 3.84 ± 0.11 | 3.83 ± 0.08 | 3.70 ± 0.10 |
| **GT2** | 4.44 ± 0.23 | 3.69 ± 0.08 | 3.71 ± 0.13 | 3.39 ± 0.22 |
|  | **Day 7** | | | |
| **GT1** | 8.97 ± 0.30 | 8.93 ± 0.23 | 8.93 ± 0.16 | 8.81 ± 0.31 |
| **GT2** | 8.97 ± 0.30 | 8.71 ± 0.34 | 8.57 ± 0.42 | 7.76 ± 0.09 |
|  | *Mist* | | | |
|  | **Day 0** | | | |
| **MT1** | 4.51 ± 0.40 | 4.06 ± 0.33 | 3.88 ± 0.47 | 3.91 ± 0.34 |
| **MT2** | 4.51 ± 0.40 | 3.76 ± 0.26 | 3.68 ± 0.33 | 3.57 ± 0.41 |
|  | **Day 7** | | | |
| **MT1** | 8.89 ± 0.08 | 8.83 ± 0.15 | 8.65 ± 0.26 | 8.44 ± 0.36 |
| **MT2** | 8.89 ± 0.08 | 8.40 ± 0.62 | 7.51 ± 0.29 | 7.43 ± 0.30 |

GT1: gaseous ozone with an input concentration of 0.62 ± 0.18 mg/L; GT2: gaseous ozone with a concentration of 5.22 ± 0.55 mg/L; MT1: mist with an input ozone concentration of 9.47 ± 0.28 mg/L; MT2: mist with an input ozone concentration of 30.27 ± 0.91 mg/L.

**Table S3 -** Mean values of lightness (*L**), color hue (*h**), and color difference (*ΔE**) of fresh-cheese model matrix treated with two concentrations of gaseous ozone (GT1 and GT2) over different exposure times, after 7 days of storage.

|  | **Exposure Time (min)** | | | |
| --- | --- | --- | --- | --- |
|  | **0 min** | **30 min** | **60 min** | **90 min** |
| **Brightness (*L**)** |  |  |  |  |
| **GT1** | 56.51 ± 0.21 d | 58.82 ± 0.70 cB | 60.25 ± 0.62 bB | 61.70 ± 1.18 aB |
| **GT2** | 56.51 ± 0.21 c | 61.95 ± 0.34 bA | 67.58 ± 2.40 aA | 69.52 ± 2.66 aA |
| **Hue Angle (*h**)** |  |  |  |  |
| **GT1** | 98.92 ± 0.30 c | 103.81 ± 5.05 bcB | 107.10 ± 0.26 b | 111.57 ± 2.91 a |
| **GT2** | 98.92 ± 0.30 c | 110.91 ± 2.35 aA | 106.01 ± 1.51 bc | 109.08 ± 2.07 ab |
| **Color Difference (*ΔE**)** |  |  |  |  |
| **GT1** | 14.81 ± 0.39 a | 12.32 ± 0.78 bA | 10.51 ± 0.56 bcA | 9.89 ± 2.52 cA |
| **GT2** | 14.81 ± 0.39 a | 9.26 ± 1.71 bB | 4.66 ± 2.46 cB | 2.49 ± 0.89 cB |

Different lowercase letters in the same row indicate a statistical difference (*p* ≤ 0.05) between exposure times within each treatment. Different uppercase letters in the column indicate a statistical difference between treatments within each exposure time. GT1: gaseous ozone with an input concentration of 0.62 ± 0.18 mg/L; GT2: gaseous ozone with a concentration of 5.22 ± 0.55 mg/L.

**Table S4** - Mean values of lightness (*L**), color hue (*h**), and color difference (*ΔE**) of fresh-cheese model matrix treated with two concentrations of ozonated mist (MT1 and MT2) over different exposure times, after 7 days of storage.

|  | **Exposure Time (min)** | | | |
| --- | --- | --- | --- | --- |
|  | **0 min** | **30 min** | **60 min** | **90 min** |
| **Brightness (*L**)** |  |  |  |  |
| **MT1** | 66.32 ± 4.82 | 67.15 ± 3.40 | 70.52 ± 3.77 | 71.61 ± 4.44 |
| **MT2** | 66.32 ± 4.82 | 72.68 ± 5.10 | 74.15 ± 1.94 | 75.61 ± 3.72 |
| **Hue Angle (*h**)** |  |  |  |  |
| **MT1** | 94.36 ± 0.88 b | 102.95 ± 0.56 ab | 110.08 ± 4.72 a | 108.25 ± 4.89 a |
| **MT2** | 94.36 ± 0.88 c | 102.19 ± 6.60 bc | 105.91 ± 10.67 ab | 112.41 ± 3.01 a |
| **Color Difference (*ΔE**)** |  |  |  |  |
| **MT1** | 12.27 ± 3.53 a | 9.66 ± 3.78 ab | 6.52 ± 4.35 ab | 5.36 ± 3.80 b |
| **MT2** | 12.27 ± 3.53 a | 6.24 ± 3.25 ab | 2.99 ± 1.63 b | 2.92 ± 1.81 b |

Different lowercase letters in the same row indicate a statistical difference (*p* ≤ 0.05) between exposure times within each treatment. MT1: mist with an input ozone concentration of 9.47 ± 0.28 mg/L; MT2: mist with an input ozone concentration of 30.27 ± 0.91 mg/L.

**Table S5 -** Fatty acid composition (%) determined by gas chromatography of untreated fresh-cheese model matrix (control) and those treated with GT2 (5.22 ± 0.55 mg/L) and MT2 (30.27 ± 0.91 mg/L) concentrations of gaseous ozone and ozonated mist, respectively, for 90 minutes**.**

|  |  | **Gas** |  |  | **Mist** |  |
| --- | --- | --- | --- | --- | --- | --- |
| **Fatty acid** |  | **Control** | **GT2** |  | **Control** | **MT2** |
| C8:0 (caprylic) |  | 1.23 ± 0.16 a | 1.16 ± 0.30 a |  | 1.03 ± 0.03 b | 1.29 ± 0.06 a |
| C10:0 (capric) |  | 2.95 ± 0.07 a | 2.64 ± 0.09 b |  | 2.65 ± 0.09 b | 3.16 ± 0.20 a |
| C12:0 (lauric) |  | 3.39 ± 0.11 a | 3.33 ± 0.12 a |  | 3.40 ± 0.06 a | 3.44 ± 0.05 a |
| C14:0 (myristic) |  | 12.33 ± 0.10 a | 12.33 ± 0.39 a |  | 12.61 ± 0.24 a | 11.75 ± 0.23 b |
| C16:0 (palmitic) |  | 39.11 ± 0.27 a | 39.48 ± 0.42 a |  | 40.29 ± 0.57 a | 39.50 ± 0.63 a |
| C16:1 (palmitoleic) |  | 2.35 ± 0.08 a | 1.91 ± 0.23 b |  | 1.99 ± 0.58 a | 2.37 ± 0.13 a |
| C18:0 (stearic) |  | 11.90 ± 0.45 a | 12.25 ± 0.78 a |  | 12.60 ± 0.30 a | 12.86 ± 0.76 a |
| C18:1n9c (oleic) |  | 24.96 ± 0.43 a | 24.81 ± 0.02 a |  | 23.61 ± 0.74 a | 23.64 ± 0.05 a |
| C18:2n6c (linoleic) |  | 1.78 ± 0.09 b | 2.13 ± 0.09 a |  | 1.82 ± 0.31 a | 2.02 ± 0.02 a |

The values (in %) are presented as mean ± standard deviation; different lowercase letters in the same row indicate significant differences (*p* ≤ 0.05) between the control and the treatment.

**Table S6** – Distribution of fatty acids (%) determined by gas chromatography in fresh-cheese model matrix: untreated control and samples treated with gaseous ozone (GT2) or ozonated mist (MT2) for 90 min.

|  |  | **Gas** | | |
| --- | --- | --- | --- | --- |
| **Fatty Acid** |  | **Control** |  | **GT2** |
| Saturated |  | 70.90 ± 0.55 a |  | 71.17 ± 0.29 a |
| Monounsaturated |  | 27.32 ± 0.50 a |  | 26.71 ± 0.21 a |
| Polyunsaturated |  | 1.78 ± 0.09 b |  | 2.13 ± 0.09 a |
| Unsaturated |  | 29.09 ± 0.54 a |  | 28.84 ± 0.30 a |
|  |  | **Mist** | | |
| **Fatty Acid** |  | **Control** |  | **MT2** |
| Saturated |  | 72.58 ± 1.27 a |  | 71.98 ± 0.10 a |
| Monounsaturated |  | 25.59 ± 1.32 a |  | 26.00 ± 0.08 a |
| Polyunsaturated |  | 1.82 ± 0.31 a |  | 2.02 ± 0.02 a |
| Unsaturated |  | 27.41 ± 1.27 a |  | 28.01 ± 0.10 a |

Values (in %) are presented as mean ± standard deviation. Different lowercase letters in the same row indicate a significant difference (*p* ≤ 0.05) between the control and treatment. GT2: gaseous ozone with a concentration of 5.22 ± 0.55 mg/L; MT2: mist with an input ozone concentration of 30.27 ± 0.91 mg/L.
